# Ancient evolution of hepadnaviral paleoviruses and their impact on host genomes

**DOI:** 10.1093/ve/veab012

**Published:** 2021-03-03

**Authors:** Spyros Lytras, Gloria Arriagada, Robert J Gifford

**Affiliations:** 1 MRC-University of Glasgow Centre for Virus Research, 464 Bearsden Rd, Bearsden, Glasgow G61 1QH, UK; 2 FONDAP Center for Genome Regulation; 3 Instituto de Ciencias Biomedicas, Facultad de Medicina y Facultad de Ciencias de la Vida, Universidad Andres Bello, Echaurren 183, Santiago, Chile

## Abstract

Hepadnaviruses (family *Hepadnaviviridae*) are reverse-transcribing animal viruses that infect vertebrates. DNA sequences derived from ancient hepadnaviruses have been identified in the germline genome of numerous vertebrate species, and these ‘endogenous hepatitis B viruses’ (eHBVs) reveal aspects of the long-term coevolutionary relationship between hepadnaviruses and their vertebrate hosts. Here, we use a novel, data-oriented approach to recover and analyse the complete repertoire of eHBV elements in published animal genomes. We show that germline incorporation of hepadnaviruses is exclusive to a single vertebrate group (Sauria) and that the eHBVs contained in saurian genomes represent a far greater diversity of hepadnaviruses than previously recognized. Through in-depth characterization of eHBV elements, we establish the existence of four distinct subgroups within the genus *Avihepadnavirus* and trace their evolution through the Cenozoic Era. Furthermore, we provide a completely new perspective on hepadnavirus evolution by showing that the metahepadnaviruses (genus *Metahepadnavirus*) originated >300 million years ago in the Paleozoic Era and have historically infected a broad range of vertebrates. We also show that eHBVs have been intra-genomically amplified in some saurian lineages, and that eHBVs located at approximately equivalent genomic loci have been acquired in entirely distinct germline integration events. These findings indicate that selective forces have favoured the accumulation of hepadnaviral sequences at specific loci in the saurian germline. Our investigation provides a range of new insights into the long-term evolutionary history of reverse-transcribing DNA viruses and shows that germline incorporation of hepadnaviruses has played a role in shaping the evolution of saurian genomes.

## 1 Background

Hepadnaviruses (family *Hepadnaviridae*) are reverse-transcribing DNA viruses that infect vertebrates. The type species—hepatitis B virus (HBV)—is estimated to infect ∼300 million people worldwide, causing substantial morbidity and mortality. Hepadnaviruses have enveloped, spherical virions and a small, circular DNA genome ∼3 kilobases (kb) in length. The genome is characterized by a highly streamlined organization incorporating extensive gene overlap—the open reading frame (ORF) encoding the viral polymerase (P) protein occupies most of the genome and typically overlaps at least one of the ORFs encoding the core (C), and surface (S) proteins.

For decades, only two hepadnavirus genera were known: genus *Orthohepadnavirus*, which infects mammalian species, and genus *Avihepadnavirus*, which infects avian species. Since 2019, however, five hepadnavirus genera are recognized (Magnius et al. 2020). The three newly defined genera include the herpetohepadnaviruses (genus *Herpetohepadnavirus*), which infect amphibians and reptiles, as well as two highly distinct groups that infect fish—the metahepadnaviruses (genus *Metahepadnavirus*) and the parahepadnaviruses (genus *Parahepadnavirus*) (Hahn et al. 2015; Dill et al. 2016; Lauber et al. 2017). Unexpectedly, phylogenetic analysis revealed that the metahepadnaviruses are more closely related to the mammalian orthohepadnaviruses than to other hepadnaviral lineages, leading to proposals that inter-class transmission of hepadnaviruses between fish and terrestrial vertebrates has occurred in the past (Dill et al. 2016; Geoghegan, Duchêne, and Holmes 2017).

Whole genome sequencing has revealed the presence of DNA sequences derived from hepadnaviruses in some vertebrate genomes. These ‘endogenous hepatitis B viruses’ (eHBVs) are thought to have originated via ‘germline incorporation’ events in which hepadnavirus DNA sequences were integrated into chromosomal DNA of germline cells and subsequently inherited as novel host alleles. Most eHBV sequences that arise in this way will be quickly purged from the gene pool via drift and natural selection. Occasionally, however, some may persist long enough to become genetically fixed in the germline of ancestral species. Fixed eHBVs are expected to remain in the germline indefinitely unless removed by macrodeletion, but in the absence of selective pressure their sequences will gradually degrade via neutral mutation.

Analysis of eHBVs has proven immensely informative with respect to the long-term evolutionary history of the *Hepadnaviridae*. eHBV sequences are in some ways equivalent to hepadnavirus ‘fossils’ in that they provide a source of retrospective information about the distant ancestors of modern hepadnaviruses. Before ancient eHBV sequences provided a means of calibrating the timeline of hepadnavirus evolution, the family was thought to have originated within the past 100,000 years. However, the discovery of ancient eHBV sequences exhibiting remarkable similarity to contemporary strains demonstrates that hepadnaviruses infected vertebrate ancestors millions of years ago, during the Mesozoic and Cenozoic Eras (Gilbert and Feschotte 2010; Katzourakis and Gifford 2010; Suh et al. 2013; Suh et al. 2014). All eHBVs identified so far derive from viruses belonging to the *Avihepadnavirus* or *Herpetohepadnavirus* genera.

Currently, the distribution and diversity of hepadnavirus-related sequences in animal genomes remains incompletely characterized. Studies have shown that multiple additional, lineage-specific eHBV insertions are present in some species (Liu et al. 2012; Cui et al. 2014; Suh et al. 2013). However, progress in characterizing these elements has been hampered by the challenges inherent in analysing large numbers of fragmentary and degenerated eHBV sequences. In this investigation, we sought to directly address these challenges and comprehensively map the distribution and diversity of eHBV sequences in vertebrate genomes. Through comparative and phylogenetic analysis of the eHBV sequences identified in our study, we derive a wide range of novel insights into the evolution of hepadnaviruses and their impact on animal genomes.

## 2 Methods

### 2.1 *Comparative analysis of hepadnavirus and eHBV sequences*

We used the GLUE software environment (Singer et al. 2018) to create a relational database that not only contains all of the data items associated with our investigation (i.e. virus genome sequences, multiple sequence alignments, genome feature annotations, and other sequence-associated data), but also represents the semantic relationships between them. Representative genome sequences for all hepadnavirus species recognized by international committee for the taxonomy of viruses (ICTV) were obtained from GenBank. Sequences of recently described hepadnaviruses not yet available in GenBank were obtained from study authors (Lauber et al. 2017).

Hepadnavirus sequences were virtually ‘rotated’ within GLUE as required to represent them within a standard coordinate space (i.e. using the same genomic start position). Using GLUE, we implemented an automated process for constructing alignments of putatively orthologous sequences. These alignments were subsequently used to derive consensus sequences representing each set of orthologs. Consensus sequences were incorporated into multiple sequence alignments (MSAs) along with hepadnavirus genome sequence. We defined a set of ‘master’ reference sequences, each of which represents a distinct clade in the hepadnavirus phylogeny. We used these sequences to create a ‘constrained alignment tree’—a data structure, implemented in GLUE (Singer et al. 2018), that links MSAs constructed at distinct taxonomic levels. eHBV coverage was calculated relative to the master reference sequence for each genus-level MSA.

### 2.2 Genome screening *in silico*

We used the database-integrated genome screening (DIGS) tool (Zhu et al. 2018) to derive a non-redundant database of loci within published whole genome sequence (WGS) assemblies that show similarity to the polypeptide gene products of hepadnaviruses. The DIGS tool is a PERL-based framework for implementing ‘database-integrated’ genome screening (DIGS). It uses the basic local alignment search tool (BLAST) program suite (Altschul et al. 1997) to perform similarity searches and the MySQL relational database management system (RDBMS) to coordinate screening and capture output data. A user-defined reference sequence library provides (1) a source of ‘probes’ for searching WGS data using the tBLASTn program, and (2) a means of classifying DNA sequences recovered screening. For the purposes of this project, we collated a reference library comprised of polypeptide sequences derived from representative hepadnavirus species (Supplementary Table S1) and previously characterized eHBVs (see Supplementary Table S2). In addition, we included polypeptide sequences derived from retroelements that show similarity to hepadnaviruses, and which could therefore be expected to produce false positive matches (Gong and Han 2018). WGS data of animal species were obtained from the National Center for Biotechnology Information (NCBI) genome database (Kitts et al. 2016). We obtained all animal genomes available as of March 2020.

Via DIGS, we generated a database of genomic sequences disclosing similarity to hepadnaviruses. We extended the core schema of this database to incorporate additional tables representing the taxonomic classifications of viruses, eHBVs and host species included in our study. We used structured query language (SQL) to interrogate the database, filtering sequences based on their similarity to reference sequences, the taxonomic properties of the closest related reference sequence, and the taxonomic distribution of related sequences across hosts. Using this approach, we categorized sequences into: (1) putatively novel eHBV elements; (2) orthologs of previously characterized eHBVs (e.g. copies containing large indels); (3) non-viral sequences that cross-matched to hepadnavirus probes (e.g. retrotransposons). Sequences that did not match to previously reported eHBVs were further investigated by incorporating them into our genus-level, genome-length MSA along with all of our reference taxa and reconstructing maximum likelihood phylogenies using RAxML (version 8) (Stamatakis 2014).

Where phylogenetic analysis supported the existence of a novel eHBV insertion, we also attempted to: (1) determine its genomic location relative to annotated genes in reference genomes; and (2) identify and align eHBV-host genome junctions and pre-integration insertion sites (see below). Where these investigations revealed new information (e.g. by confirming the presence of a previously uncharacterized eHBV insertion), we updated our reference library accordingly. This in turn allowed us to reclassify all of the putative eHBV loci in our database and group sequences more accurately into categories. By iterating this procedure, we progressively resolved the majority of eHBV sequences identified in our screen into groups of orthologous sequences derived from the same initial germline incorporation event (Supplementary material). eHBV elements were given unique IDs using a systematic approach developed for endogenous retroviruses (ERVs) (Gifford et al. 2018).

### 2.3 *Genomic analysis*

We used EMBOSS getorf (Rice, Longden, and Bleasby 2000) to extract all open reading frames starting with a start codon of length > 300 bp for the consensus endogenous viral element (EVE) sequences of each group (avi, herpeto, meta). No ORFs other than the known P, C, and S were consistently found in more than one EVE within groups. A motif search was performed for the few short unknown ORFs that were detected, using the Motif Search online tool (https://www.genome.jp/tools/motif/) against motif libraries Pfam (El-Gebali et al. 2019), NCBI-CDD (Marchler-Bauer et al. 2005), and Prosite Pattern. No protein motifs could be detected with an E-value threshold of 0.01, indicating that these short ORFs are unlikely to have any real function. Based on this analysis, we conclude that there are no notable features of genome evolution to further report on the manuscript. However, sampling of new extant Avi, Herpeto, or Metahepadnavirus genomes in the future might allow for further investigation of the EVE sequences reported here.

To confirm that the eHBV elements identified in our study were distinct from those previously reported (i.e. they derive from a distinct germline incorporation event), we investigated the locus surrounding each putatively novel eHBV. We extracted DNA sequences flanking eHBV insertions and used BLASTn to identify the homologous insertion sites in species listed in the ENSEMBL genome browser. To assess potential presence of transposable elements (TE) around eHBVs of interest (Fig. 3a), we extracted the 5 kb sequences flanking the eHBV coordinates from the respective genome assemblies, adjusting for reverse complementarity. These sequences were analysed for TE presence using HMMER [Eddy 2009] against the Dfam HMM profile library [Hubley et al. 2016].

### 2.4 *Insertion dating and plotting of evolutionary timelines*

The minimum ages of eHBV insertions reported in this study were inferred by identifying the most distantly related pair of host species sharing a particular eHBV and applying the estimated species divergence dates and confidence intervals (CI) as reported in TimeTree (Kumar et al. 2017). A plot showing the timeline of hepadnavirus evolution was created using Adobe Illustrator™ software. Time calibrations for phylogenetic trees were derived from our own analyses and those provided by Lauber et al. (Lauber et al. 2017).

## 3 Results

### 3.1 Endogenization of hepadnaviruses is unique to saurian species

**Figure 1.**
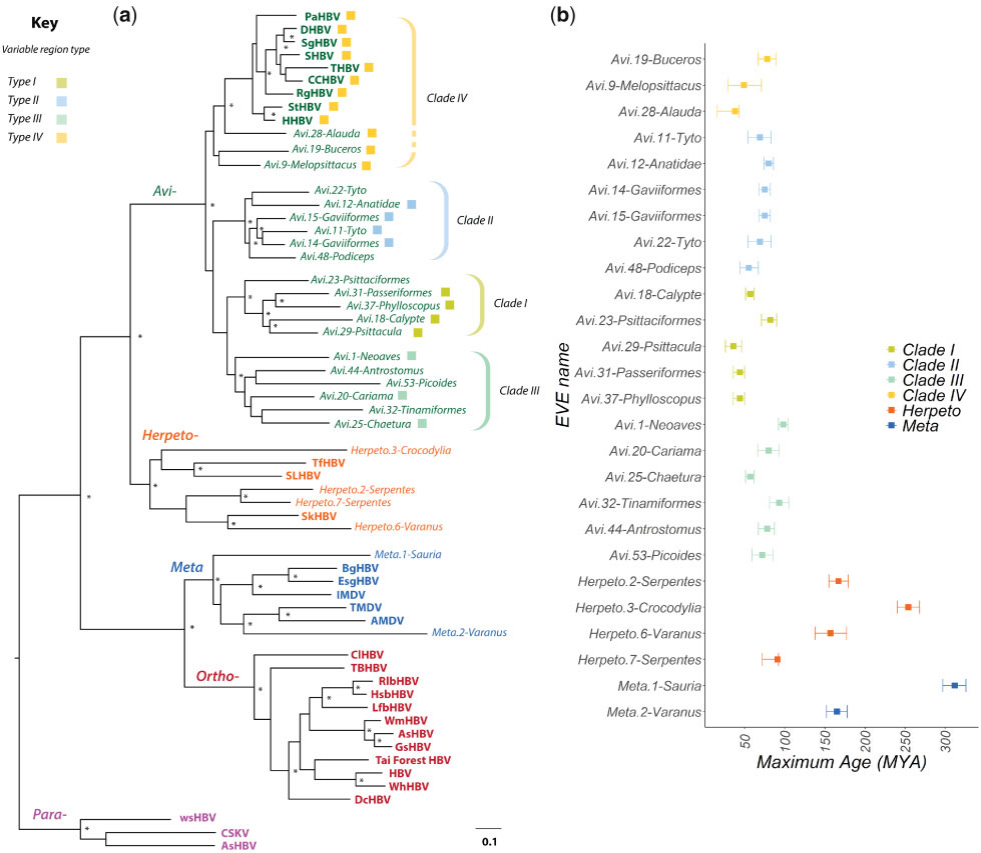
Recovery of paleovirus sequences reveals the evolutionary history of hepadnaviruses. Panel (a) shows a maximum likelihood phylogeny constructed using codons 355–440 and 500–781 of the polymerase (P) protein (Hepatitis B virus coordinates, GenBank reference sequence accession number: NC_003977). The phylogeny is rooted on the Parahepadnavirus genus, based on the basal position of this genus in trees constructed using the Nackednaviruses as an outgroup (Supplementary Fig. S1). Virus names are shown in bold. IDs of endogenous hepatitis B viruses (eHBVs) are shown in italic. Taxon label colours correspond to viral genera as follows: purple = Parahepadnavirus; blue = Metahepadnavirus; red = Orthohepadnavirus; orange = Herpetohepadnavirus; green = Avihepadnavirus. Virus name abbreviations are as shown in Supplementary Table S1. Brackets to the right indicate subclades within the avihepadnavirus genus. The dashed bracket for Clade IV denotes that grouping of eHBV elements 9, 19, and 28 does not have high support here, but is supported by other phylogenetic evidence. Coloured squares next to avihepadnavirus taxon labels variable region ‘type’ as indicated by the key. Some eHBV sequences do not span this region and thus cannot be assigned. Asterisks indicate nodes with bootstrap support >=70, based on 1,000 replicates. The scale bar shows evolutionary distance in substitutions per site. The plot in panel (b) shows the window in geological time during which we estimated various eHBV insertions to have been generated, based on their distribution across vertebrate taxa. Abbreviations: Mya = Million years ago.

We screened WGS data of 1,220 animal species (included 415 invertebrates and 805 vertebrates) for endogenous hepadnaviral elements (eHBVs). Via screening we identified 930 (Table 1, Supplementary material) sequences that disclosed a high degree of similarity to the polypeptide gene products of contemporary hepadnaviruses. We found that *bona fide* eHBV elements are only present in the genomes of saurian species. Furthermore, reconstruction of the phylogenetic relationships between eHBVs and contemporary hepadnaviruses revealed that saurian genomes contain a broader diversity of eHBV elements than previously recognized, with elements derived metahepadnaviruses being present, as well as elements derived from the *Avihepadnavirus* and *Herpetohepadnavirus* genera (Fig. 1a, Supplementary Fig. S1a). We estimate that the 930 eHBV sequences identified in our study correspond to at least 55 distinct hepadnavirus eHBV ‘lineages’, with each lineage being derived from a distinct germline incorporation event. Included among this set are forty-three insertions derived from avihepadnaviruses (ten have been reported previously), eight derived from herpetohepadnaviruses (five have been reported previously), and six derived from metahepadnaviruses. All previously described eHBV elements were identified in our screen.

**Table 1.** EHBV loci detected in vertebrate genomes.

eHBV element ID^a^	Citation	Virus clade^b^	Num. Species^c^	Num. Seqs^d^	Flanking genes^e^		Min age^f^	Max age^g^
					Upstream	Downstream		
**Metahepadnavirus**								
Meta.1-Sauria			27	*27*	*KLF8*	*ENSAC*	280 (273–286)	312 (297–326)
Meta.2-Varanus			1	1	*NPVF*	*NFE2L3*	–	165 (152–178)
Meta.3-Paroedura			1	1	*n/k*	*n/k*	–	96 (83–98)
Meta.4-Pelusios			1	1	*LCP1*	*RUBCNL*	–	99 (70–128)
Meta.5-Pelusios			1	1	*KCNA5*	*NTF3*	–	99 (70–128)
Meta.6-Sphenodon			1	1	*n/k*	*n/k*	–	252 (241–263)
**Herpetohepadnavirus**								
Herpeto.1-Serpentes^h^	(Suh et al. 2014)	Snake	9	9	*TSHZ1*	*ZNF516*	62 (49–74)	91 (72–92)
Herpeto.2-Serpentes^h^	(Suh et al. 2014)	Snake	10	10	*NPFFR2*	*FTH1*	62 (49–74)	167 (155–179)
Herpeto.7-Serpentes		Snake	3	3	*ATP2A3*	*ZZEF1*	9.2 (5.8–18.8)	91 (72–92)
Herpeto.8-Serpentes		Snake	5	5	*OBI1*	*RBM26*	62 (49–74)	91 (72–92)
Herpeto.6-Varanus		Lizard	1	1	**WSCD2**	**WSCD2**	–	*157 (138*–*177)*
Herpeto.3-Crocodylia^h^	(Suh et al. 2014)	Croc.	1	1	*NUP210*	*IQSEC1*	44 (25–64)	254 (240–268)
Herpeto.4-Crocodylia^h^	(Suh et al. 2014)	Croc.	1	1	*SORT1*	*PPIL1*	26.7 (22–29)	254 (240–268)
Herpeto.5-Testudines^h^	(Suh et al. 2014)	Turtle	18	18	*GBE1*	*ROBO1*	184 (161–206)	254 (240–268)
**Avihepdnavirus**								
Avi.18-Calypte		I	1	1	***ENSSCUG00000017518***	***ENSSCUG00000017518***	–	57 (51–62)
Avi.23-Psittaciformes		I	11	71	*n/a*	*n/a*	49 (29–71)	82 (71–90)
Avi.29-Psittacula		I	1	1	***KIDINS220***	***KIDINS220***	–	36 (26–46)
Avi.31-Passeriformes^i^		I	7	7	*TIMM21*	*NETO1*	38 (16–43)	44 (36–50)
Avi.37-Phylloscopus		I	2	2	*EPHA3*	*ZNF654*	1.2	44 (36–50)
Avi.49-Psittaciformes		I	14	14	***GRID2***	***GRID2***	38 (30–50)	82 (71–90)
Avi.52-Melopsittacus		I	1	1	*LUZP2*	*ANO3*	–	38 (14–55)
Avi.11-Tyto		II	1	1	*NAV3*	*E2F7*	–	69 (54–83)
Avi.12-Anatidae		II	5	9	***CCDC58***	***CCDC58***	30 (26–35)	80 (74–86)
Avi.14-Gavia		II	1	1	*TAS1R3*	*DVL1*	–	75 (68–82)
Avi.15-Gavia		II	1	1	***TMEM182***	***TMEM182***	–	75 (68–82)
Avi.22-Tyto		II	1	1	*MYBL2*	*PTPRT*	–	69 (54–83)
Avi.24-Apaloderma		II	1	1	***CDH23***	***CDH23***	–	72 (59–85)
Avi.27-Phalacrocoracidae		II	4	233	*n/a*	*n/a*	9.6	73 (59–87)
Avi.35-Calypte		II	1	1	*CYB5A*	*TIMM21*	–	57 (51–62)
Avi.46.Psittaciformes		II	10	10	*FMN1*	*RYR3*	49 (29–71)	82 (71–90)
Avi.48-Podiceps		II	1	1	*MATN1*	*PTPRU*	–	55 (44–67)
Avi.1-Neoaves^h^	(Suh et al. 2013)	III	110	111	***FRY***	***FRY***	85 (77–94)	98 (92–104)
Avi.20-Cariama		III	1	1	*THBS1*	*KATNBL1*	–	80 (66–93)
Avi.25-Chaetura		III	1	1	*KCNV1*	*ENSTGUG00000027711*	–	57 (51–62)
Avi.32-Tinamiformes		III	2	2	*OLFM4*	*n/k*	49 (37–62)	93 (81–105)
Avi.34-Leptosomus		III	1	1	***LRRC7***	***LRRC7***	–	70 (58–82)
Avi.42-Passeriformes^i^		III	5	5	***HECA***	***HECA***	48 (33–50)	82 (75–90)
Avi.44-Antrostomus		III	1	1	*KCNV1*	*CSMD3*	–	78 (67–87)
Avi.53-Picoides		III	1	1	*TRIB2*	*LPIN1*	–	72 (59–85)
Avi.28-Alauda		IV	1	1	*EPHA6*	*NSUN3*	–	38 (16–43)
Avi.4-Passeriformes^h^^,^^i^	(Suh et al. 2013)	IV	9	9	***CDH23***	***CDH23***	38 (16–43)	82 (75–90)
Avi.5-Passeriformes^h^^,^^i^	(Suh et al. 2013)	IV	5	5	*LMO3*	*MGST1*	38 (16–43)	82 (75–90)
Avi.6-Estrildinae^h^	(Suh et al. 2013)	IV	2	2	*FOXD3*	*ATG4C*	10.1(8.7–11.6)	11.8 (11–14)
Avi.8-Australiaves^h^	(Suh et al. 2013)	IV	18	28	***ATP2B2***	***ATP2B2***		82 (71–90)
Avi.38-Passeriformes		IV	8	8	*TGIF2*	*AAR2*	38 (16–43)	82 (75–90)
Avi.9-Melopsittacus^h^	(Liu et al. 2012)	IV	1	1	***CD109***	***CD109***	–	49 (29–71)
Avi.19-Buceros		IV	1	1	*PCDH18*	*PCDH10*	–	78 (67–89)
Avi.7-Passeriformes^h^^,^^i^	(Suh et al. 2013)		6	6	*TMEM132E*	*LIG3*	38 (16–43)	82 (75–90)
Avi.13-Paleognathea			8	8	*ENSDNVG00000017897*	*BCKDHB*	93 (81–105)	111 (105–118)
Avi.16-Turaco			1	1	*TMEM8B*	*ENSACUG00000013925*	–	74 (63–85)
Avi.21-Paleognathea			8	8	*LMCD1*	*GRM7*	93 (81–105)	111 (105–118)
Avi.26-Psittaciformes			7	8	***NELL1***	***NELL1***	38 (14–55)	82 (71–90)
Avi.30-Anatidae			5	5	*ENSACDG00005009727*	*CCDC58*	30 (26–35)	80 (74–86)
Avi.39-Passeriformes^i^			9	9	***FXN***	***FXN***	38 (30–50)	82 (75–90)
Avi.41-Psittaciformes			4	4	*PHAX*	*KLF4*	38 (30–50)	82 (71–90)
Avi.43-Gallirallus			1	1	***DEND4A***	***DEND4A***	–	64 (52–75)
Avi.45-Psittaciformes			8	8	***RNF38***	***RNF38***	38 (30–50)	82 (71–90)

a eHBV elements were given unique IDs using a systematic approach, following a convention developed for endogenous retroviruses (Gifford et al. 2018). Each is assigned a unique identifier (ID) constructed from three components. The first component (not shown here) is the classifier ‘eHBV’ denoting an endogenous hepadnaviral element. The second component comprises: (1) the name of the hepadnavirus genus the element derived from and; (2) a numeric ID that uniquely identifies a specific integration locus, or for multi-copy lineages, a unique founding event. The final component denotes the taxonomic distribution of the element.

b Virus taxonomic groups below genera level are shown where known.

c Number of species in which the insertion/lineage was identified.

d Total number of orthologs/duplicated identified in screen.

e Names of annotated genes flanking each insertion. Intronic insertions are shown in bold. EMSEMBL gene IDs are shown for genes that do not yet have names. n/k = not known; n/a = not applicable to multi-copy lineages;

f Minimum age as determined via orthology and based on divergence times provided by TimeTree (Kumar et al. 2017);

g Maximum age based on presence of empty insertion site in a sister clade, or other evidence (see Methods).

h Previously reported elements.

i eHBVs distributed across unranked clades.

**Figure 2.**
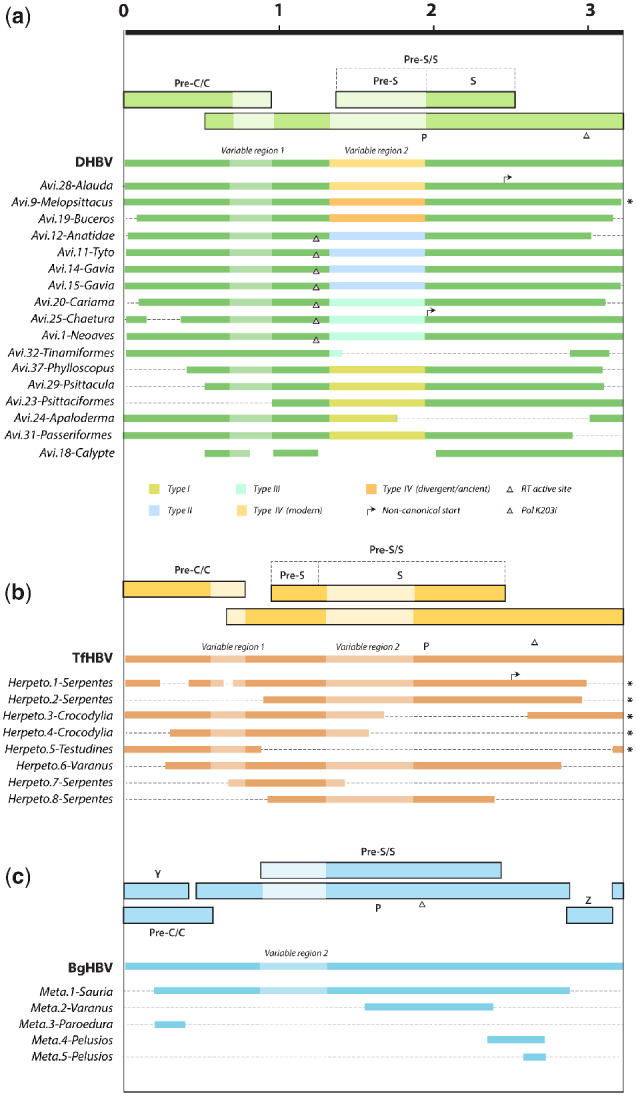
Consensus genomic organization of endogenous hepadnaviral element (eHBV) insertions identified in this study. eHBV structures are shown relative to the genomes of prototype virus species from the corresponding genus. Virus names are shown in bold, eHBV names are shown in italic. Thinner bars represent nucleic acid sequences. Thicker bars represent open reading frames in viral sequence. Asterisks indicate sequences that have been reported previously. Scale bar indicates sequence length in kilobases. Key shows relationships between symbols/shading and genome features. Abbreviations: DHBV, duck hepadnavirus; TfHBV, Tibetan frog hepadnavirus; BgHBV, bluegill hepadnavirus; Pre-C/C, Pre-Core/Core; P, Polymerase; Pre-S/S, Pre-Surface/Surface.

Relatively large numbers of avihepadnavirus-derived eHBVs were identified in avian genomes (Table 1), most of which represent only short, sub-genomic fragments. However, we identified seventeen that represented complete, or near-complete viral genomes (Fig. 2). We also identified previously unreported, herpetohepadnavirus-derived eHBVs in the genomes of a lizard (superorder Lepidosauria) and in snakes (order Serpentes). The novel snake elements were closely related to those previously reported in snake genomes (Suh et al. 2013) (Fig. 1a, Supplementary Fig. S1), while the lizard element was identified in the Komodo dragon (*Varanus komodoensis*). *Herpeto.6-Varanus* was found to cluster robustly with skink hepatitis B virus (SkHBV) (Lauber et al. 2017).

Notably, metahepadnavirus-like eHBV elements were identified in a wide range of saurian species, including birds, turtles, a lizard—the ocelot gecko (*Paroedura pictus*)—and the tuatara (*Sphenodon punctatus*). By contrast, herpetohepadnavirus-derived elements were only identified in reptiles, and avihepadnavirus-derived elements were only detected in birds.

### 3.2 Several distinct avihepadnavirus lineages have circulated among birds during their evolution

Phylogenetic reconstructions demonstrate the presence of at least four distinct clades (I–IV) within the *Avihepadnavirus* genus (Fig. 1). Clade IV contains a mixture of extant avihepadnaviruses and eHBV insertions, while the remaining three clades are comprised exclusively of eHBVs. Notably, all four *Avihepadnavirus* clades are highly divergent from one another in ‘variable region 2’ (which spans most of the Pre-S protein and includes regions that encode receptor-binding functions (Glebe and Urban 2007)), but within each clade these regions are relatively well conserved (Fig. 2, Supplementary Fig. S2a). The order of ancestral branching among *Avihepadnavirus* clades is unclear—in phylogenies constructed using highly conserved regions of the P gene and rooted on herpetohepadnaviruses, none is clearly basal or derived relative to the others (data not shown). Notably, however, clade IV and clade II share a conserved, synapomorphic character: the insertion of a valine (V) or isoleucine (I) residue in the P protein, between positions 203 and 204 (Fig. 2, Supplementary Fig. S2b). This shared, conserved character indicates that these two clades are more closely related to one another than they are to other hepadnaviruses—at least in the region around the synapomorphy. Overall, germline incorporation events involving each of the four avihepadnavirus clades seem to have occurred throughout the evolution of birds, with some occurring prior to major divergences in the avian tree, and others being confined to specific avian species or subgroups (Table 1, Fig. 1b).

Near-complete insertions derived from clade I were identified in rose-necked parakeets (*Avi.29-Psittacula*) and in Anna’s hummingbird (*Calypte anna*) as well as two distinct elements in songbirds (order Passeriformes). Among the two songbird elements, one was found only in warblers (*Avi.37-Phylloscopus*) while another (*Avi.37-Passeriformes*) was found in five distinct families within the superfamily Passeroidea, establishing that it integrated into the passeroid germline >38 Mya (CI: 16–43 Mya). We also identified clade I-derived elements in parrots that represent only fragments of a hepadnavirus genome. These elements, which appear to have been intra-genomically amplified (discussed below) and include some elements that are orthologous across all parrots (order Psittaciformes), indicate that germline incorporation occurred >49 Mya (CI: 29–71 Mya) prior to the divergence of the kea (*Nestor notabilis*) from other parrot lineages (Kumar et al. 2017).

Clade II contains sequences derived from ducks (family Anatidae), red-throated divers (*Gavia stellata*), and barn owls (*Tyto alba*). The insertion in ducks was incorporated >30 Mya (CI 26–35 Mya), prior to the divergence of mallards (*Anas platyrhynchos*) and ruddy ducks (*Oxyura jamaicensis*). Notably, multiple, genome-length eHBV elements derived from this lineage were often identified in the same species or species group. For example, multiple, clade II-derived eHBVs were identified in both the *Tyto* (*Avi.11* and *Avi.22*) and *Gavia* (*Avi.14* and *Avi15*) germlines. However, in-depth analysis of these sequences shows that each derives from distinct germline incorporation events. Not only are they located in entirely distinct genomic loci (Table 1), they also show higher divergence in the variable regions of their genome than in other more conserved regions (Supplementary Fig. S3)—this is consistent with them being separated by multiple rounds of viral replication, rather than neutral divergence following an intra-genomic duplication process. Notably, the greatest extent of divergence was observed in the regions of the genome that encode receptor-binding functions.

Clade III includes the ‘*Avi.1-Neoaves*’ element (previous names include eAHBV-FRY (Lauber et al. 2017) and eZHBVc (Suh et al. 2013)), which is the first avihepadnavirus-derived eHBV element to be reported, and is also the oldest. It is orthologous across the Neoaves clade, which includes all avian species except the paleognathes (infraclass Paleognathae) and fowl (Galloanserae; ducks, chickens, and allies). We identified additional eHBVs derived from this lineage in a broad range of avian groups. Notably, clade I-derived insertions are present in the paleognathe germline: the genomes of white-throated and Chilean tinamous contain orthologous eHBVs demonstrating that clade I avihepadnaviruses circulated in paleognathe birds >49 Mya (CI 37–62 Mya). In addition, we identified clade I-derived insertions in order Trogoniformes represented by the bar-tailed trogon (bar-tailed trogon), in clade Strisores, represented by the swift (*Chaetura pelagica*), and in clade Australiaves, represented by the red-legged seriema (*Cariama cristata*). This broad distribution is consistent with the demonstrably ancient origins of this lineage.

All clade IV-derived eHBVs group basal to the exogenous avihepadnaviruses, which cluster together as a derived, crown group within this clade. We identified a full-length insertion in the Eurasian skylark (*Alauda arvensis*) genome that shows a higher level of relatedness to modern hepadnaviruses than does any previously reported eHBV (Fig. 1a). Notably, *eHBV-Avi.28-Alauda* was the only avihepadnavirus-derived eHBV element found to exhibit similarity to modern avihepadnaviruses in the variable region of the genome (Fig. 2, Supplementary Fig. S2a). Some phylogenetic trees support the inclusion of eHBV elements previously reported in the budgerigar genome (Liu et al. 2012), and a newly identified element identified in the genome of the rhinoceros hornbill (*Buceros rhinoceros*), within clade IV (Supplementary Fig. S1g).

### 3.3 *Avian genomes contain multi-copy eHBV lineages*

**Figure 3.**
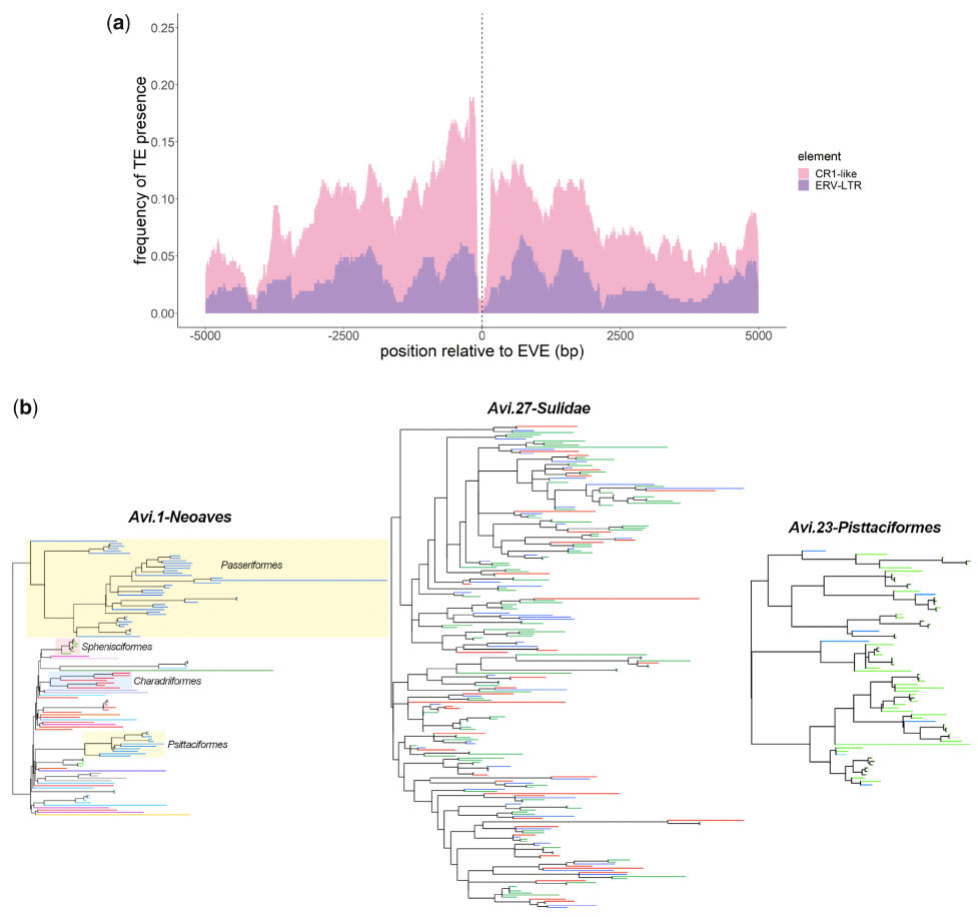
Genomic characteristics of multi-copy eHBV lineages. (a) The plot shows the frequency with which specific transposable element (TE) sequences were detected in 5 kb regions flanking 307 distinct members of the multi-copy eHBV lineage *Avi.27-Phalacrocoracidae*. Sequences were analysed for the presence of transposable elements (TE) using HMMER (Eddy 2009) against the Dfam HMM profile library (Hubley et al. 2016). Based on their descriptions, TEs detected in flanking sequences were divided into two categories: (1) related to the chicken repeat 1 group of retrotransposons (CR1) (shown in pink); (2) related to ERV long terminal repeat (LTR) (shown in purple); (b) Maximum likelihood (ML) phylogenies of multi-copy eHBV element lineages. All phylogenies were constructed using nucleotide sequence data, under the GTR+I model of evolution, Support for ML trees was assessed via 1,000 non-parametric bootstrap replicates. (1) *Avi.1.Neoaves* (Supplementary Fig. S4); (2) *Avi.27.Phalacrocoracidae* (Supplementary Fig. S5); (3) *Avi.23.Psittaciformes* (Supplementary Fig. S6). The terminal branches of all tips in the phylogenies are coloured based on their hosts’ taxonomic classification. The *Avi.1.Neoaves* phylogeny is labelled on the order level, *Avi.27.Phalacrocoracidae* on the genus level, and *Avi.23.Psittaciformes* on the family level. The order names of clear clades with multiple representatives are annotated on the *Avi1.Neoaves* phylogeny.

In addition to genome-length sequences, avian genomes contain multiple eHBV elements that represent only fragments of an avihepadnaviral genome. Furthermore, some avian lineages contain expanded sets of highly related eHBVs. Most strikingly, we identified >300 copies of a highly duplicated eHBV element in cormorants and shags (order Suliformes). This lineage, named *Avi.27-Phalacrocoracidae*, appears to be derived from a single germline incorporation event involving an ancient, clade II avihepadnavirus (Supplementary Fig. S1g), and is comprised of fragments spanning a short region at the 3′ terminal end of the *pol* gene. Investigation of *Avi.27* elements revealed that the vast majority are flanked on both sides by transposable element (TE) sequences (Fig. 3a), suggesting this multi-copy lineage may have arisen in association with TE activity (i.e. integration into a TE led to an eHBV-derived sequence being mobilized). The lack of clear and precise TE-eHBV boundaries in these loci could reflect that: (1) the mobile element driving replication was a mosaic of other elements (e.g. similar to the HARLEQUIN element found in the human genome (Vargiu et al. 2016)), or (2) there have been more insertions and rearrangements associated with the history of this lineage. Unfortunately, we cannot differentiate between these possibilities based on the available data.

Phylogenies indicate that the initial germline incorporation event that gave rise to this multi-copy eHBV lineage predates the diversification of the four cormorant species in which it was identified, as evidenced by the presence of multiple, multi-species sub-clusters in phylogenies (see Fig. 3b) and the presence of multiple orthologous integration sites (data not shown).

We also identified apparently intra-genomically amplified, avihepadnavirus-derived eHBV elements in the genomes of parrots. In this case, the amplified elements appear to derive from an ancient clade I avihepadnavirus. Although the elevated eHBV copy number found in certain avian orders reflects intra-genomic amplification, it is nonetheless clear that the rate of germline incorporation is significantly higher in birds than in any other vertebrate group. We characterized eHBV loci in saurian genomes by identifying the nearest annotated genes upstream and downstream of EVE integration sites (Table 1). Excluding integrations that occurred as a result of intra-genomic amplification, we estimate that at least 57 distinct germline incorporation events—each involving a distinct hepadnavirus progenitor—have occurred during avian evolution.

### 3.4 *eHBV elements are enriched at specific loci in the saurian germline*

**Figure 4.**
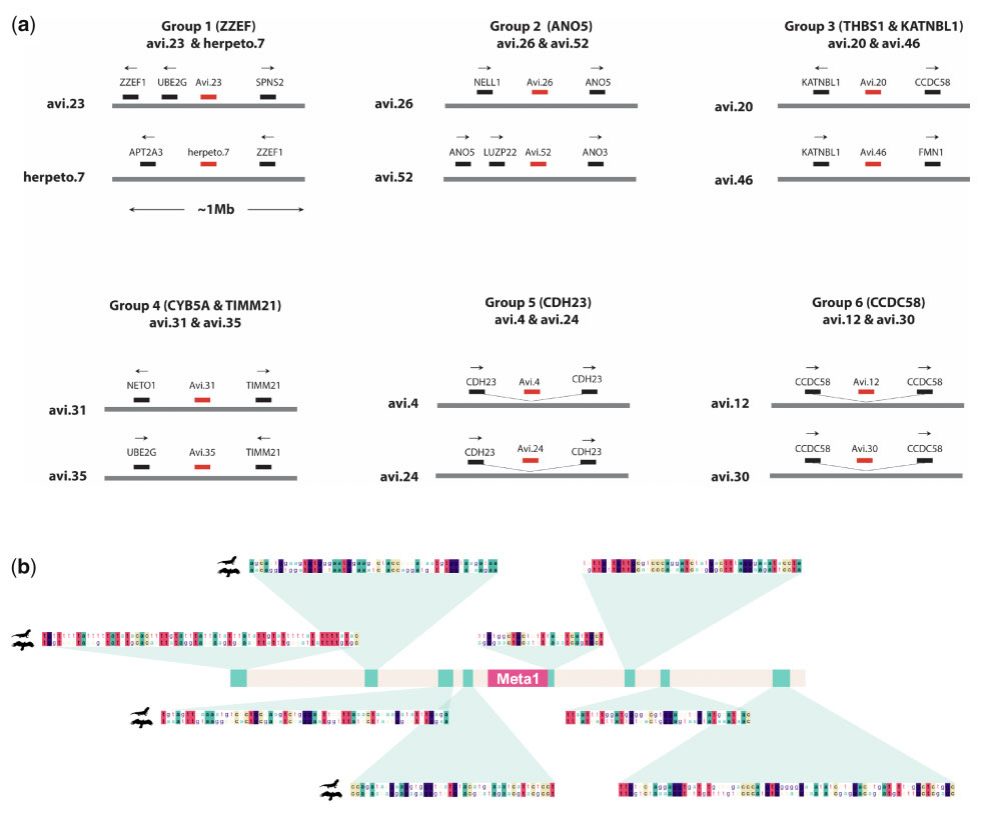
Characteristics of selected eHBV loci. (a) Loci containing multiple fixed eHBV elements. Schematic representation of six genomic loci at which pairs of eHBV insertions derived from distinct eHBV lineages occur adjacent to one another. Grey bars represent genomic DNA. Black bars represent genes or exons with arrows showing the direction of transcription. Red bars represent eHBV elements. (b) Schematic representation of the *Sphenodon punctatus* copy of the *Meta.1-Sauropsida* (Meta1) genomic region including 1 kb flanking regions to each side of the EVE. The sequence homology between the *S. punctatus* (top) and the *A. chrysaetos canadensis* (bottom) genome is highlighted in representative subregions around the orthologous EVE, labelled in green.

We investigated the genes flanking eHBV loci using ENSEMBL, revealing that several are inserted into intronic regions of genes (Table 1). Strikingly, this analysis also identified several pairs of elements that are fixed at distinct yet broadly equivalent genomic sites. We identified six cases in which eHBV elements that appear to derive from distinct germline incorporation events have been fixed at nearly equivalent genomic loci (Table 2). Most involved avihepadnaviruses in avian genomes, but in these cases the two eHBV elements usually derived from distinct clades within the *Avihepadnavirus* genus. We also identified one case in which an avihepadnavirus-derived element found in the avian germline (specifically, one member of the multi-copy *Avi.23* lineage) is located at the same approximate genomic position as a herpetohepadnavirus-derived element (*Herpeto.7*) in snake genomes (Fig. 4a). Open accessible chromatin at these parts of the genome might be responsible for the apparent repeated integrations at these loci. In fact, genes in all six loci presented in Fig. 4a (ZZEF1, ANO5, KATNBL1, TIMM21, CDH23, CCDC58) are constitutively expressed in all embryonic developmental stages in chickens according to the EBI Expression Atlas (Papatheodorou et al. 2019) suggestive of open chromatin.

**Table 2.** Pairs of apparently distinct EHBV insertions at adjacent loci.

Pair name	First member		Second member	
	Name	Genus (Clade)^a^	Name	Genus (Clade)^a^
1 (ZZEF)	Avi.23-Psittaciformes	Avi- (I)	Herpeto.7-Serpentes	Herpeto- (Snake)
2 (ANO5)	Avi.52-Melopsittacus	Avi- (I)	Avi.26-Psittaciformes	Avi- (NK)
3 (CDH23 intronic)	Avi.20-Cariama	Avi- (III)	Avi.46-Psittaciformes	Avi- (II)
4 (CYB5A & TIMM21)	Avi.35-Calypte	Avi- (II)	Avi.31-Passeriformes	Avi- (I)
5 (FBXO15-NETO1)	Avi.24-Apaloderma	Avi- (II)	Avi.4-Passeriformes	Avi- (IV/V)
6 (CCDC58 intronic)	Avi.12-Anatidae	Avi- (I)	Avi.30-Anatidae	Avi- (NK)

a Avi-, Avihepadnavirus; Herpeto-, Herpetohepadnavirus.

### 3.5 *Metahepadnaviruses circulated in the late Paleozoic era*

Most of the metahepadnavirus-like elements identified in our screen were comprised of short fragments ∼300–500 nucleotides (nt) in length. However, a group of orthologous, metahepadnavirus-derived eHBV elements identified in birds contained some copies that spanned a near-complete genome (Fig. 2). Furthermore, in-depth investigation of this insertion demonstrates that it is clearly orthologous across a diverse range of avian species, including eagles, implying that it originated >83 Mya (CI: 77–90 Mya). Even more remarkably, our investigation revealed that an element identified in the tuatara is likely a member of the same group of orthologous insertions. This implies that germline incorporation of the element—labelled *eHBV*-*Meta.1-Sauria—*occurred prior to the divergence of the Lepidosauromorpha and Archosauromorpha ∼282 Mya (Kumar et al. 2017). Given that: (1) we found evidence for independent insertion and fixation of eHBVs at approximately equivalent genomic loci (see above) and (2) due to deletion of large regions of terminal eHBV sequence, none of the eHBV-genomic DNA junctions are precisely equivalent on either side of the avian and lepidosaur orthologs, this finding has to be interpreted with caution. However, in each of the pairs of insertions that we propose to have been independently integrated (see Fig. 4a), insertions are only located at approximately similar genomic sites. By contrast, the genomic flanks upstream and downstream of the tuatara and avian elements show a strikingly similar arrangement of conserved non-coding sequences (Fig. 4b). Since DNA loss is characteristic of Saurian evolution (Kapusta et al. 2017), the equivalent genomic region could presumably have been deleted in other major clades descending from the Lepidosaur–Archosaur ancestor.

## 4 Discussion

Our investigation provides a range of new insights into the deep evolutionary history of hepadnaviruses and their impact on animal genomes. Firstly, we show that germline incorporation of hepadnavirus sequences is unique to saurians, despite the fact that hepadnaviruses are known to infect a much broader range of vertebrate groups. It is unclear why germline incorporation is restricted to saurian hosts, but access to germline cells is likely to be a key underlying factor. The relatively high level of genome invasion might be related to specific aspects of transmission and replication in this particular host–virus system (i.e. avi- or herpetohepadnavirus infections in saurian hosts)—particularly as they relate to vertical transmission. Studies of avihepadnavirus infections in domestic ducks show that virus is normally transmitted via vertical transmission *in ovo* and this may be the case in other avian species (Jilbert, Reaiche-Miller, and Scougall 2019). Conceivably, herpeto-/avi- hepadnaviruses could have come to rely more on vertical transmission via infection of germline cells than other hepadnaviral genera, perhaps in relation to certain aspects of the saurian reproduction system (e.g. internal fertilization and the shelled egg), and this provided increased opportunity for germline incorporation to occur. Selective pressures at the host level, particularly the potential for population bottlenecks during the evolution and radiation of avian species, may also have played a role.

We show that the diversity of hepadnavirus sequences contained within saurian genomes is much higher than has previously been appreciated. In particular, the high frequency of germline incorporation in avian lineages allowed for a far more extensive characterization of avihepadnavirus diversity. Our analysis identified four major subclades within the *Avihepadnavirus* genus, each of which has a relatively broad distribution among avian species. So far, all exogenous virus species have only been identified in one of these clades. However, all appear to have circulated throughout a large part, if not most of the Cenozoic Era. However, due to lack of genome coverage across avian species, we were only able to obtain an approximate timeline of evolution for each of the four avihepadnaviral lineages. Conceivably, the existence of the four clades might reflect the historical compartmentalization of avian subpopulations (e.g. due to geographic isolation) during certain periods of their evolution. Currently, we do not have a sufficient level of precision to infer any association between the ancestral distribution of avihepadnavirus strains and the evolutionary history of specific bird lineages. However, the upcoming publication of data from the avian 10K genomes project (Zhang 2015) should allow a more precise dating of eHBV integration times.

**Figure 5.**
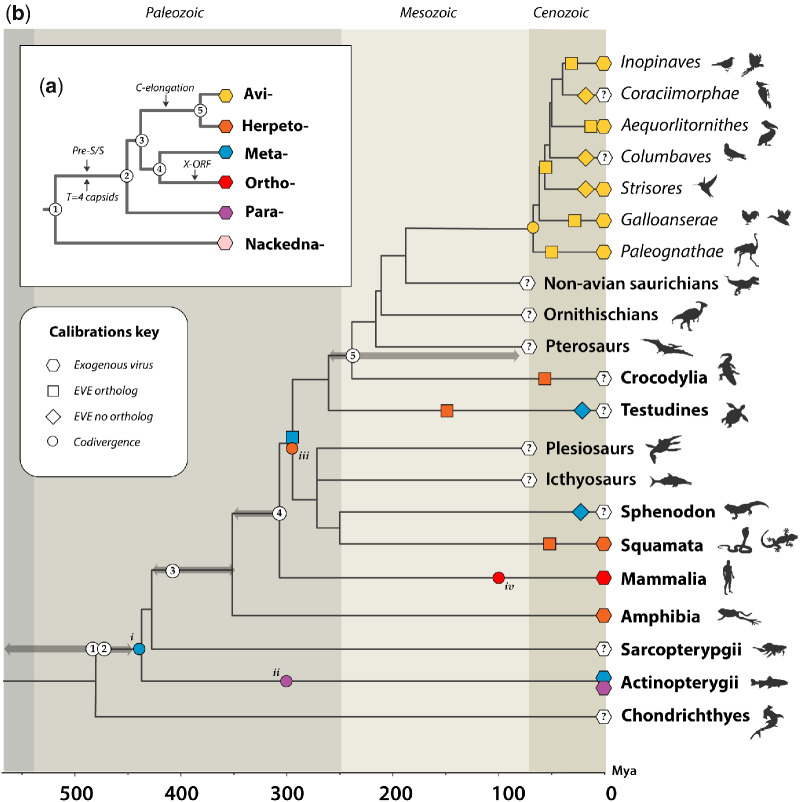
Timeline of hepadnavirus evolution. The inset panel (a) shows a schematic phylogeny depicting the established evolutionary relationships between hepadnaviral genera, with black arrows indicating the most parsimonious periods of major evolutionary innovations (after Lauber (Lauber et al. 2017)). Internal nodes are numbered in reference to the time-calibrated phylogeny of vertebrates that is shown in panel (b). In panel (b), geological eras are indicated by background shading and the scale bar shows time in millions of years before present. Colours indicate hepadnaviral genera as shown in panel (a). Shapes on branches indicate four distinct types of time calibration as shown in the key. Note that the identification of EVEs that lack orthologous copies (indicated by diamonds) does not allow minimum ages to be inferred, but nonetheless indicates the presence of hepadnavirus in the ancestral members of a given lineage. Numbers in white circles show the putative locations of nodes on the hepadnavirus tree in relation to the timeline of vertebrate evolution. Calibrations based on the assumption of codivergence are represented by circles, as follows; (1) metahepadnaviruses—found in fish and saurians; (2) parahepadnaviruses—found in all teleosts (Lauber et al. 2017); (3) herpetohepadnaviruses (based on the assumption that TfHBV originated via inter-class transfer from saurians to amphibians); (4) orthohepadnaviruses—present in major lineages of placental mammals (Laurasiatheria, Euarchontoglires) as viruses. Grey arrows flanking numbered nodes on the host tree indicate time range in which the corresponding virus divergence could have occurred. Abbreviations: C, Core; S, Surface; EVE, endogenous viral element; Mya, Million years ago.

We identified several eHBV insertions derived from metahepadnavirus-like viruses, as well as from avi- and herpetohepadnaviruses. These are, to the best of our knowledge, the first metahepadnavirus EVEs to be reported and accordingly they provide a completely new perspective on the evolution of the genus. Remarkably, analysis of the broader genomic landscape surrounding one insertion (*eHBV-Meta.1-Sauria*) indicates that it was inserted into the germline an estimated 280 Mya (CI: 273–286 Mya) (Fig. 5a). This makes *eHBV-Meta.1-Sauria* the oldest EVE described to date, and the first example of an EVE derived from a virus that circulated in the Paleozoic Era (541–252 million years ago). Recent debate has focused on the relationship between short and long-term substitution rate estimates in virus genomes (Holmes and Duchêne 2019; Simmonds, Aiewsakun, and Katzourakis 2019). Dividing the branch length separating HBV and the node connecting meta- and orthohepadnaviruses (node 4 in Fig. 5) by 300 Mya (the time calibration provided by *eHBV-Meta.1-Sauria*) provides an approximate estimate of 2.59 × 10^−9^ substitutions per site per year for the long-term rate of substitution rate in conserved regions of the *pol* gene. This matches closely with recently published estimates of long-term substitution rates inferred for polymerases and other highly conserved genes in several virus families, including hepadnaviruses (Membrebe et al. 2019; Simmonds, Aiewsakun, and Katzourakis 2019).

Metahepadnaviruses have only been identified very recently (Geoghegan, Duchêne, and Holmes 2017), and along with the parahepadnaviruses (genus Parahepadnavirus) they are the first hepadnaviruses known to infect fish. Whereas the parahepadnaviruses are only distantly related to other hepadnavirus genera, phylogenetic analysis unexpectedly revealed that the *Metahepadnavirus* genus groups as a relatively close sister taxon to the mammalian orthohepadnaviruses in phylogenies (Geoghegan et al. 2018). This has been widely interpreted as evidence of cross-species transmission between fish and mammals (Dill et al. 2016; Geoghegan, Duchêne, and Holmes 2017). However, the discovery that metahepadnaviruses infected ancestral vertebrates challenges these conclusions. We identify metahepadnavirus-derived eHBV sequences derived in a turtle and a lizard (Table 1, Figs 1 and 2) showing that these viruses not only circulated in saurian ancestors, but also infected the reptile descendants of these organisms. Combined with ancient age of the genus implied by the eHBVs identified in our study, this allows for a simpler explanation of contemporary hepadnavirus distributions, wherein the *Hepadnaviridae* diverge into distinct ‘Meta-Ortho’ and ‘Herpeto-Avi’ lineages prior to the divergence of fish and tetrapods and then subsequently co-diverged (broadly speaking) with their host groups (see Fig. 5). As outlined elsewhere, this pattern of evolution does not necessarily preclude zoonotic transmission of related hepadnaviruses (e.g. viruses from the same genus) between related groups of hosts, and phylogenetic analysis does seem to suggest that inter-class transmission of herpetohepadnaviruses has occurred between reptiles and amphibians. In general, however, the greater the taxonomic distance between hosts, the less likely a zoonotic jump is to be successful (Holmes 2009).

We show that eHBVs have been intra-genomically amplified in suliforme birds—most likely in association with transposable element (TE) activity—and a large number of these insertions have been fixed. We have previously reported a similar phenomenon for endogenous circoviral elements in carnivore genomes (Dennis et al. 2019), and it has also been described for ERVs in primates—for example, hominid genomes contain SVA (SINE-R, VNTR, and Alu) elements that contain a portion of HERV-K(HML2) (Wang et al. 2005). More broadly, it seems that the sequences of certain mammalian apparent LTR retrotransposon (MaLR) lineages, such as the HARLEQUIN elements found in the human genome (Vargiu et al. 2016), comprise complex mosaics of ERV fragments. Possibly, the capture of EVE sequences offers a selective advantage to TE lineages. Alternatively, TE sequences containing hepadnavirus-derived DNA might, for some reason, be more likely to be fixed.

Consistent with the idea that germline incorporation of hepadnavirus sequences might, in some cases, be favoured by selection at the level of the host, we identified multiple examples of loci containing multiple fixed eHBV elements, each derived from a distinct germline colonization event (Fig. 4a). In principle, the enrichment of eHBVs at specific loci could reflect natural selection—i.e. eHBVs were integrated randomly into genomes, and those integrated at specific loci were selected over time—for example, due to a favourable influence on gene regulation as has been widely reported for TEs and ERVs in animal genomes (Chuong, Elde, and Feschotte 2017; Enriquez-Gasca, Gould, and Rowe 2020). However, it could also reflect the preferential integration of hepadnaviruses into these loci (e.g. because they are accessible in embryonic cells).

Comparative studies of eHBVs have been greatly hampered by the challenges associated with analysing these sequences, which are often highly degraded by germline mutation. This may explain why—despite the fact that it has been clear for some time that additional, lineage-specific eHBV insertions are present in some vertebrate species—progress in characterizing and analysing novel eHBV sequences has been quite slow. This likely reflects the manifold challenges encountered in identifying and characterizing eHBVs. Complicating factors include the hepadnavirus genome structure: the overlapping reading frames and circular genome, both of which can make recovering the ancestral structure of integrated eHBVs less straightforward than it is for other kinds of endogenous viral element. Additional complications arise due to the intra-genomic duplication and re-arrangement of eHBV sequences, and the fact that the hepadnaviral polymerase, which occupies a large proportion of the hepadnavirus genome, shares distant similarity with the reverse transcriptase genes encoded by certain retroelements. While all of these contingencies can be dealt with in one way or another, this is usually done in an *ad hoc* way that makes it difficult for other investigators to recapitulate or build on the work done by previous investigators. In this study, we sought to directly address these challenges by using a novel data-orientated approach. This allowed us to publish our findings in the form of an online resource that not only contains all of the data items associated with our investigation (i.e. virus genome sequences, multiple sequence alignments, genome feature annotations, and other sequence-associated data), but also represents the semantic relationships between these data items. Furthermore, via the GLUE engine (a platform-independent software environment) (Singer et al. 2018), it provides the means to recapitulate all of the analyses performed in our study.

## Supplementary Material

veab012_Supplementary_Data
