# Supplementary material for: Ancient evolution of hepadnaviral paleoviruses and their impact on host genomes
Source: Virus Evol. 2021 Mar 3;7(1):veab012. doi: 10.1093/ve/veab012 (PMC7955980; doi:10.1093/ve/veab012)
Supplement: veab012_Supplementary_Data [file veab012_supplementary_data.zip › table s1.pdf]

Table S1. Hepadnavirus genome sequences used as references in this study.

Sequence ID	Virus name	Host scientific name	Host common name
**Avihepadnavirus**			
NC_001344	Duck hepatitis B virus	*Anas platyrhynchos*	Domestic duck
NC_001486	Heron hepatitis B virus	*Ardea cinerea*	Grey heron
NC_016561	Parrot hepatitis B virus	*Psittacula krameri*	Rose-ringed parakeet
NC_005888	Ross's goose hepatitis B virus	*Chen rossii*	Ross's goose
NC_005950	Snow goose hepatitis B virus	*Anser caerulescens*	Snow goose
NC_005890	Sheldgoose hepatitis B virus	*Chloephaga poliocephala*	Ashy-headed sheldgoose
NC_035210	Tinamou hepatitis B virus	*Eudromia elegans*	Elegant crested tinamou
AJ441111	Crane hepatitis B virus	*Balearica regulorum*	Grey crowned crane
AJ251934	Stork hepatitis B virus	*Ciconia ciconia*	White stork
**Herpetohepadnavirus**			
NC_030446	Tibetan frog hepadnavirus	*Nanorana parkeri*	Tibetan frog
SLHBV-1	Spiny lizard herpetohepadnavirus	*Sceloporus adleri*	Spiny lizard
SkHBV	Skink herpetohepadnavirus	*Saproscincus basiliscus*	Skink
**Metahepadnavirus**			
NC_030445	Bluegill hepadnavirus	*Lepomis macrochirus*	Bluegill
MH716822	Eastern sea garfish hepatitis B virus	*Hyporhamphus australis*	Eastern sea garfish
AMDV	Astatotilapia metahepadnavirus	*Astatotilapia sp*	Astatotilapia
TMDV	Tetra metahepadnavirus	*Astyanax mexicanus*	Mexican tetra
IMDV	Icefish metahepadnavirus	*Chionodraco hamatus*	Crocodile icefish
**Orthohepadnavirus**			
NC_003977	Hepatitis B virus	*Homo sapiens*	Human
NC_028129	Woolly monkey hepatitis B virus	*Lagothrix lagothricha*	Woolly monkey
NC_001484	Ground squirrel hepatitis virus	*Spermophilus beecheyi*	California ground squirrel
NC_024444	Horseshoe bat hepatitis B virus	*Rhinolophus alcyone*	Horseshoe bat
NC_020881	Long-fingered bat hepatitis B virus	*Miniopterus fuliginosus*	Long-fingered bat
NC_024443	Roundleaf bat hepatitis B virus	*Hipposideros cf. ruber*	Roundleaf bat
NC_024445	Tent-making bat hepatitis B virus	*Hipposideros cf. ruber*	Tent-making bat
NC_004107	Woodchuck hepatitis virus	*Marmota monax*	Woodchuck
MH307930	Domestic cat hepadnavirus	*Felis catus*	Domestic cat
MH484442	Shrew hepatitis B virus	*Crocidura lasiura*	Ussuri white-toothed shrew
MK620908	Tai Forest hepadnavirus	*Philantomba maxwellii*	Maxwell's duiker
U29144	Arctic ground squirrel hepatitis B virus	*Spermophilus parryii*	Arctic ground squirrel
**Parahepadnavirus**			
CSKV	Coho salmon parahepadnavirus	*Oncorhynchus kisutch*	Coho salmon
NC_027922	White sucker hepadnavirus	*Catostomus commersonii*	White sucker
MH716821	Australasian snapper hepadnavirus	*Pagrus auratus*	Australasian snapper
