# Supplementary material for: Ancient evolution of hepadnaviral paleoviruses and their impact on host genomes
Source: Virus Evol. 2021 Mar 3;7(1):veab012. doi: 10.1093/ve/veab012 (PMC7955980; doi:10.1093/ve/veab012)
Supplement: veab012_Supplementary_Data [file veab012_supplementary_data.zip › table s2.pdf]

Table S2. Previously reported eHBV elements used in this investigation

Sequence ID (this study)	Previous names	Citation
ehbv-avi.1-neoaves-con	Zebrafinch EVE, eZHBVc, eAHBV-FRY	[1-3]
ehbv-avi.2-estrildidae	eZHBVm	[3]
ehbv-avi.3-passeriformes	eZHBV01	[3]
ehbv-avi.4-passeriformes	eZHBV02	[3]
ehbv-avi.5-passeriformes	eZHBV03	[3]
ehbv-avi.6-passeriformes	eZHBV04	[3]
ehbv-avi.7-passeriformes	eZHBV05	[3]
ehbv-avi.8-australiaves	eZHBV0e	[3]
ehbv-avi.9-melopsittacus	eBHBV1	[4]
ehbv-avi.10-melopsittacus	eBHBV2	[4]
ehbv-herpeto.1-serpentes-con	eSNHBV1	[5]
ehbv-herpeto.2-serpentes-con	eSNHBV2	[5]
ehbv-herpeto.3-crocodilia	eCRHBV1	[5]
ehbv-herpeto.4-crocodilia	eCRHBV2	[5]
ehbv-herpeto.5-testudines	eTHBV	[5]

References

1. Katzourakis, A. and R.J. Gifford, *Endogenous viral elements in animal genomes.* PLoS Genet, 2010. **6**(11): p. e1001191.
2. Gilbert, C. and C. Feschotte, *Genomic fossils calibrate the long-term evolution of hepadnaviruses.* PLoS Biol, 2010. **8**(9).
3. Suh, A., et al., *The genome of a Mesozoic paleovirus reveals the evolution of hepatitis B viruses.* Nat Commun, 2013. **4**: p. 1791.
4. Liu, W., et al., *The first full-length endogenous hepadnaviruses: identification and analysis.* J Virol, 2012. **86**(17): p. 9510-3.
5. Suh, A., et al., *Early mesozoic coexistence of amniotes and hepadnaviridae.* PLoS Genet, 2014. **10**(12): p. e1004559.
